# Fasciculoventricular bypass tracts: Electrocardiographic and electrophysiologic features

**DOI:** 10.1002/joa3.12355

**Published:** 2020-05-08

**Authors:** Dimitrios Asvestas, George Bazoukis, Panagiotis Mililis, Stelios Dragasis, Athanasia Megarisiotou, Konstantinos Vlachos, Antonios Sideris, Michael Efremidis, Konstantinos P. Letsas

**Affiliations:** ^1^ Arrhythmia Unit Laboratory of Cardiac Electrophysiology “Evangelismos” General Hospital of Athens Athens Greece

**Keywords:** accessory pathway, fasciculoventricular, preexcitation

## Abstract

Fasciculoventricular accessory pathways are rare variants of preexcitation. The differential diagnosis of fasciculoventricular accessory pathways from other preexcitation variants can be challenging. Based on two cases, we discuss the specific electrocardiographic and electrophysiologic features of fasciculoventricular bypass tracts.

## Graphical abstract

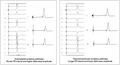

## 1 INTRODUCTION

Fasciculoventricular (FV) accessory pathways (AP) are uncommon preexcitation variants.^1^, ^2^ The differential diagnosis of FV APs from anteroseptal atrioventricular APs and nodoventricular (NV)/nodofascilular (NF) APs can be challenging.^1^, ^2^ Based on two cases, we discuss the specific electrocardiographic and electrophysiologic features of FV bypass tracts.

## 2 CASE REPORT 1

**FIGURE 1:**
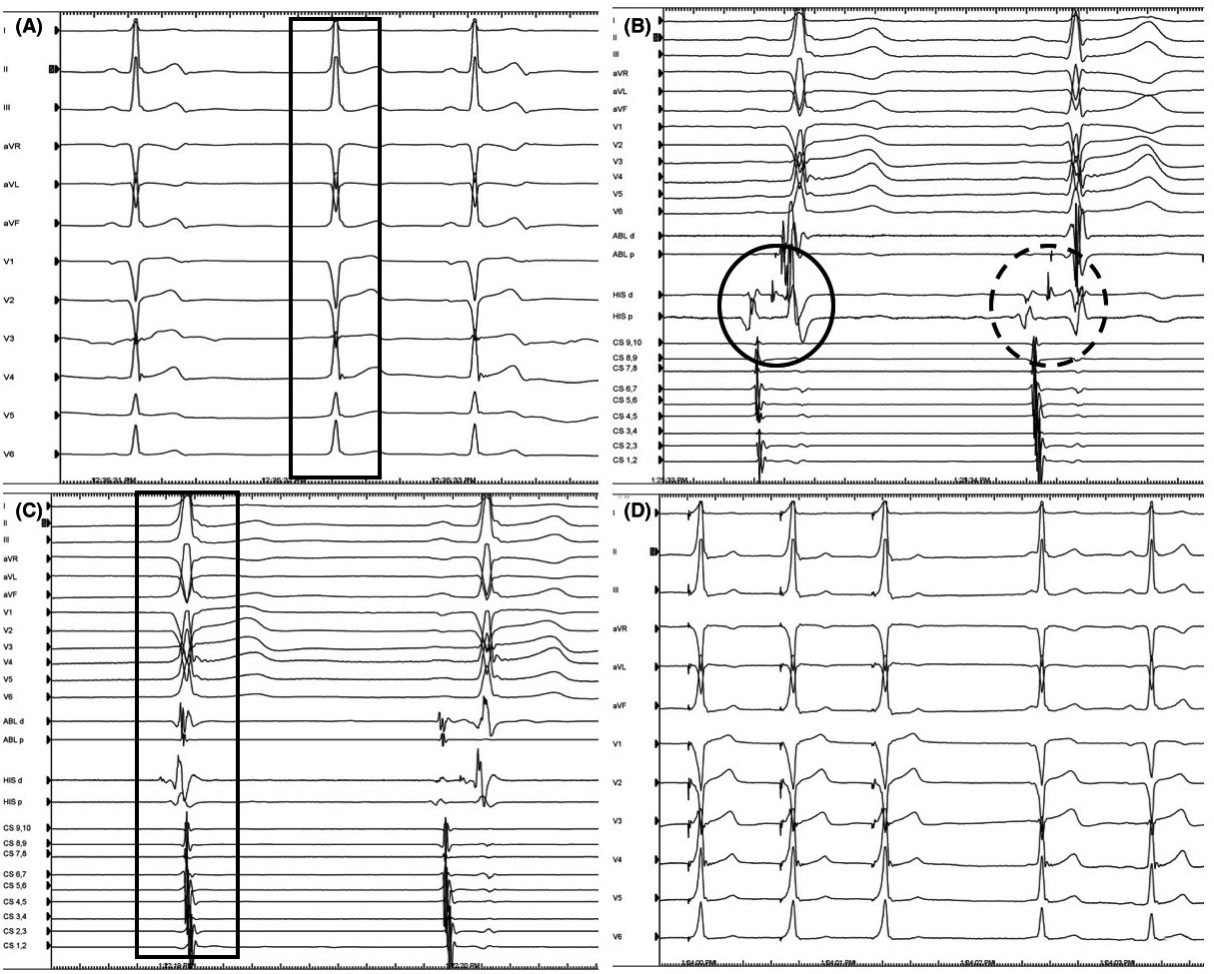
A, Baseline ECG showing minimal preexcitation (absence of septal q waves). A junctional beat with the same ECG configuration is marked; B, Spontaneous loss of preexcitation with HV prolongation (dashed cycle). The distal (ABL p bipole) and proximal His (HIS d bipole) electrograms are recorded; C, Junctional beat (marked beat) with identical preexcitation pattern and the same HV interval (HIS d bipole) as in sinus rhythm; D, Para‐Hisian pacing produced a near perfect match regarding the degree of preexcitation. ABL, ablation catheter; HIS, His bundle catheter; CS, coronary sinus catheter

A 31‐year‐old male was referred for an electrophysiologic study (EPS) because of overt preexcitation on 12 lead electrocardiogram (ECG). He never complained for palpitations or tachycardia. He had no history of structural heart disease and the transthoracic echocardiogram was normal. The QRS duration was 105 milliseconds with normal frontal plane axis, normal PR interval with minimal preexcitation (absence of septal q waves), and precordial transition (R/S wave ratio > 1) in lead V_3_ (Figure 1A). The differential diagnosis included the presence of anteroseptal AP or NV/NF AP or FV AP. During EPS, the AH interval was normal and the HV interval was 28 milliseconds Sudden prolongation of the HV interval and loss of preexcitation occurred either spontaneously or following atrial pacing at slow rates, confirming a long effective refractory period of the AP (Figure 1B). Interestingly, during EPS, junctional beats with identical preexcitation pattern were recorded, while the HV interval remained short and unchanged, suggesting the presence of an infranodal AP (Figure 1A and C). Para‐Hisian pacing produced a near perfect match with the baseline ECG (Figure 1D). Programmed atrial pacing with or without isoproterenol failed to induce any supraventricular tachycardias. Based on these findings, the presence of a FV AP was established. No ablation was performed and the patient was discharged and remains asymptomatic.

## 3 CASE REPORT 2

**FIGURE 2:**
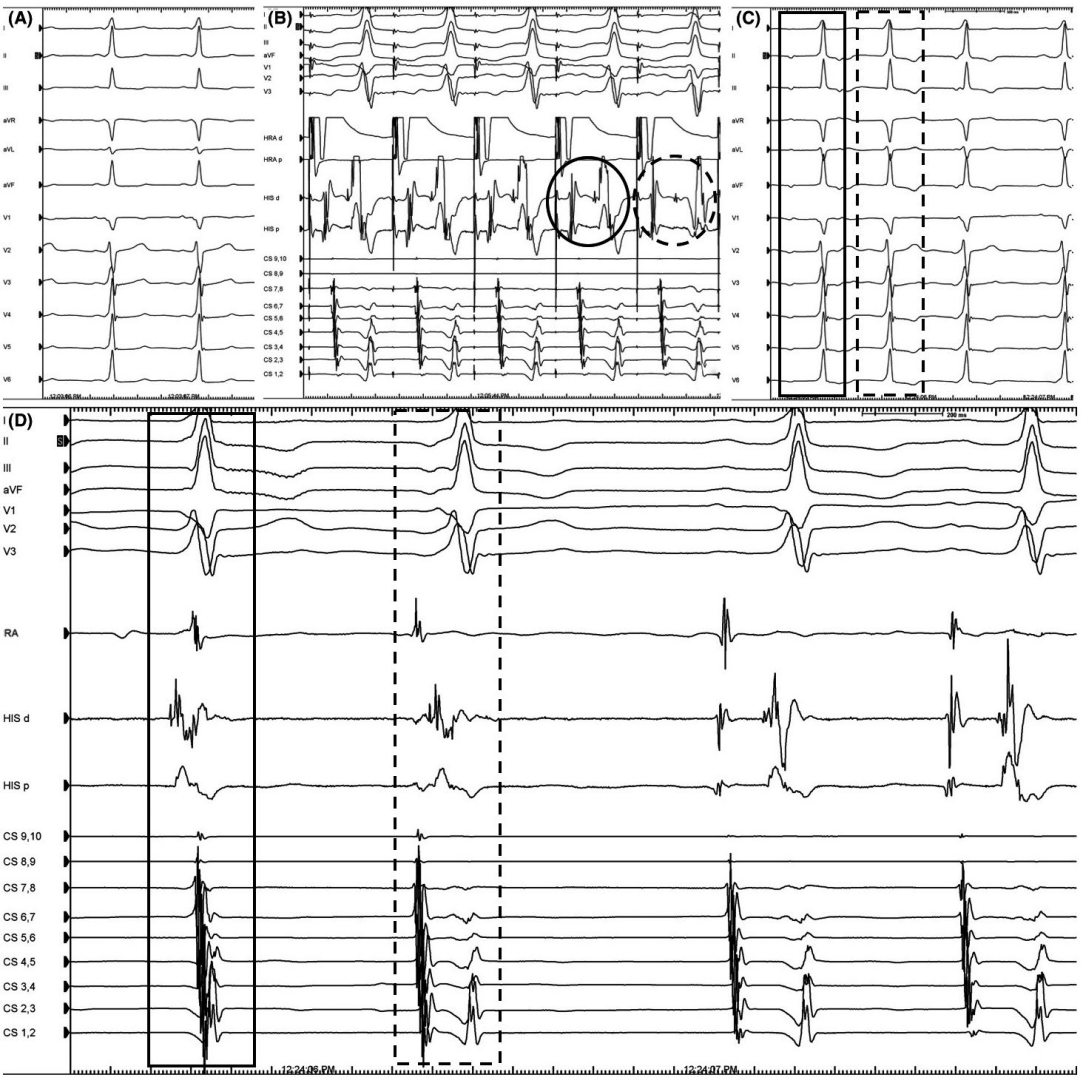
A, Baseline ECG showing minimal preexcitation; B, Loss of preexcitation with HV interval prolongation (dashed cycle, HIS d bipole) during incremental atrial pacing from the right atrium (HRA d bipole); C, Different patterns of AV conduction (solid marked beat) as well as a junctional beat (dashed marked beat) with the same degree of preexcitation were noted; D, Intracardiac electrograms during a junctional beat (solid marked beat) and a low right atrial beat (dashed marked beat) with identical preexcitation pattern and the same HV interval (HIS d bipole). HRA, high right atrium catheter; RA, right atrium catheter; HIS, His bundle catheter; CS, coronary sinus catheter

A 17‐year‐old female was referred for an EPS owing to overt preexcitation on 12‐lead ECG. She never complained for palpitations or tachycardia. The ECG showed a wide QRS (>120 milliseconds) with normal frontal plane axis, a short PR interval with minimal preexcitation (positive delta waves in leads II, aVF, V4‐6), and precordial transition in lead V_3_ (Figure 2A). The echocardiographic study was normal. The differential diagnosis included the presence of anteroseptal AP or NV/NF AP or FV AP. During EPS, the baseline HV interval was short, at 15 milliseconds, and stayed fixed during incremental atrial pacing until AP refractoriness was reached (Figure 2B). Of note, the degree of preexcitation remained the same during incremental atrial pacing (Figure 2B). The administration of 18mg adenosine induced different atrioventricular (AV) conduction patterns (prolongation of PR interval), AV block as well as junctional rhythm without producing any change in the configuration of the QRS complex (Figure 2C and D), an event that favored the diagnosis of FV AP. Programmed atrial pacing with or without isoproterenol failed to induce any supraventricular tachycardias. No ablation was attempted and the patient remains asymptomatic until today.

## 4 DISCUSSION

**FIGURE 3:**
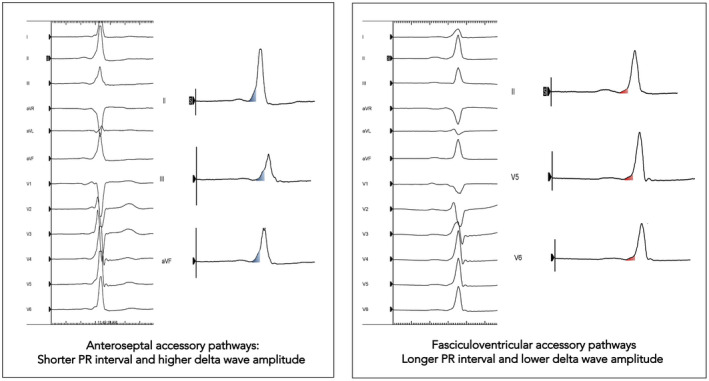
Differences in the surface ECG between anteroseptal (left panel) and FV (right panel) APs based on the study of O'Leary et al^4^ Anteroseptal APs display shorter PR intervals and higher delta wave amplitudes (blue triangle) compared to FV (red triangle) APs. AP, accessory pathway; FV, fasciculoventricular

FV APs are extremely rare preexcitation variants which have distinctive ECG and electrophysiological features. These fibers take off from the His bundle and the fascicles to the right ventricle.^1^, ^2^ They have only antegrade and nondecremental conducting properties.^1^, ^2^ The ECG is characterized by normal frontal plane axis, similar to an anteroseptal APs, a subtle preexcitation, and a normal PR interval. R/S transition in the precordial leads is mostly recorded in lead V_2_.^3^ Anteroseptal APs display significantly higher delta wave amplitudes (4.8 ± 2.0 mm vs 1.9 ± 1.3 mm), shorter PR intervals (94.6 ± 12.5 milliseconds vs 106.8 ± 13.2 milliseconds), and longer QRS intervals (133.6 ± 19.0 milliseconds vs 118.7 ± 24.7 milliseconds) compared to FVFs.^4^ In the later study, the delta wave amplitude was the only independent predictor of WPW syndrome.^4^ Figure 3 shows these ECG differences between anteroseptal and FVAPs.

Regarding the electrophysiologic features of FV APs, the HV interval (H‐delta) is always short (10‐35 ms).^1^, ^2^ During incremental atrial pacing, the progressive increase of AH interval along with a fixed HV interval and the same degree of preexcitation are suggestive of FVP.^1^, ^2^ On the contrary, a NF or NV is not totally an infranodal structure, as it bypasses only a part of the AV node which displays decremental conduction properties. Therefore, the preexcitation degree may change with rapid atrial pacing and the HV interval may decrease even to negative values. Block at the FV AP during incremental atrial pacing results in normal HV interval, and produces a narrow QRS complex without preexcitation. The presence of FV APs is further suggested when the administration of adenosine prolongs the PR interval or the AH interval without changing the degree of preexcitation.^1^, ^2^ The presence of preexcited junctional beats favors the diagnosis of FV APs.^1^, ^2^ However, junctional beats arising from the transitional zone/atrionodal connection may display preexcitation in the setting of NF/NV APs.^5^ A true His extrasystole producing the same preexcitation degree is pathognomonic of FVP.^5^ High‐output para‐Hisian pacing may reproduce an identical preexcitation morphology, indicating the presence of a FVP.^1^, ^2^, ^5^ FV APs should be differentiated from anteroseptal (para‐Hisian) or NF/NV APs, since the latter are implicated in supraventricular tachycardias and catheter ablation near the AV node should be attempted. The electrophysiologic characteristics of typical AV (such as an anteroseptal AP), NF/NV and FV APs are depicted in Table 1. Although FV APs are not implicated in clinical tachycardias, they have been associated with structural abnormalities and sudden cardiac death. In the setting of PRKAG2, gene mutations have a high incidence of syncope, ventricular hypertrophy, atrial arrhythmias, sinus bradycardia, and complete AV block.^6^

**TABLE 1:** Electrocardiographic and electrophysiologic characteristics of typical atrioventricular (orthodromic reciprocating tachycardia), nodoventricular (NV)/nodofascicular (NF) and fasciculoventricular (FV) accessory pathways (APs)

	Atrioventricular AP	NV/NF APs	FV AP
Baseline preexcitation	Yes	Minimal or none	Minimal or none
Participation in tachycardia	Yes	Yes	No
1:1 AV relationship during tachycardia	Obligatory	Not obligatory	No participation in tachycardia
Incremental atrial pacing			
Stim A‐V interval	Relatively fixed	Increase	Increase
H‐V interval	Decrease	Decrease	Fixed
Preexcitation	Increase	Increase	Fixed
Junctional beats	Not preexcited	Possibly preexcited if originating proximally	Preexcited
Adenosine response	Full preexcitation	AV block	AV block with preexcited beats

In conclusion, the diagnosis of FV APs is essential, mainly for two reasons. First, in the setting of a supraventricular tachycardia, the recognition of the bystander nature of these APs is extremely important in order to avoid unnecessary catheter ablation and inadvertent damage of the AV node. Second, subjects with FV APs require further investigation in order to exclude a PRKAG2 gene mutation phenotype which has been associated with sudden cardiac death. Although challenging, prompt differentiation of FVs from anteroseptal APs is therefore extremely important. Even in the absence of clinical tachycardias, the diagnosis of FV APs should be established and their association with genetic syndromes related to structural heart disease and sudden cardiac death should be excluded.

## CONFLICT OF INTERESTS

The authors declare no conflict of interests for this article.
